# Perceived Quality of Life Is Related to a Healthy Lifestyle and Related Outcomes in Spanish Children and Adolescents: The Physical Activity, Sedentarism, and Obesity in Spanish Study

**DOI:** 10.3390/nu15245125

**Published:** 2023-12-16

**Authors:** Marina Ródenas-Munar, Margalida Monserrat-Mesquida, Santiago F. Gómez, Julia Wärnberg, María Medrano, Marcela González-Gross, Narcís Gusi, Susana Aznar, Elena Marín-Cascales, Miguel A. González-Valeiro, Lluís Serra-Majem, Susana Pulgar, Marta Segu, Montse Fitó, Silvia Torres, Juan Carlos Benavente-Marín, Idoia Labayen, Augusto G. Zapico, Jesús Sánchez-Gómez, Fabio Jiménez-Zazo, Pedro E. Alcaraz, Marta Sevilla-Sánchez, Estefanía Herrera-Ramos, Helmut Schröder, Cristina Bouzas, Josep A. Tur

**Affiliations:** 1 Research Group on Community Nutrition and Oxidative Stress, University of Balearic Islands-IUNICS, 07122 Palma de Mallorca, Spain; m.rodenas@uib.cat (M.R.-M.); margalida.monserrat@uib.es (M.M.-M.);; 2 Centro de Investigación Biomédica en Red Fisiopatología de la Obesidad y la Nutrición (CIBEROBN), Institute of Health Carlos III, 28029 Madrid, Spainmarcela.gonzalez.gross@upm.es (M.G.-G.);; 3 Health Research Institute of Balearic Islands (IdISBa), 07120 Palma, Spain; 4 Gasol Foundation Europe, 08830 Sant Boi de Llobregat, Spain; 5 CIBER de Epidemiología y Salud Pública (CIBERESP), Instituto de Salud Carlos III, 28049 Madrid, Spain; 6 Cardiovascular Risk and Nutrition Research Group (CARIN), Hospital del Mar Institute for Medical Research, 08003 Barcelona, Spain; 7 GREpS, Health Education Research Group, Nursing and Physiotherapy Department, University of Lleida, 25003 Lleida, Spain; 8 EpiPHAAN Research Group, Universidad de Málaga—Instituto de Investigación Biomédica de Málaga (IBIMA), 29071 Málaga, Spain; jc.benaventemarin@gmail.com; 9 ELIKOS Group, Institute for Sustainability and Food Chain Innovation (IS-FOOD), Department of Health Sciences, Public University of Navarre, 31006 Pamplona, Spain; 10 ImFINE Research Group, Department of Health and Human Performance, Universidad Politécnica de Madrid, 28223 Madrid, Spain; azapico@edu.ucm.es; 11 Physical Activity and Quality of Life Research Group (AFYCAV), Faculty of Sport Sciences, University of Extremadura, 10003 Cáceres, Spain; narcis.gusi@gmail.com (N.G.);; 12 PAFS Research Group, Faculty of Sports Sciences, University of Castilla-La Mancha-Toledo Campus, 45004 Toledo, Spainfabio.jimenez@uclm.es (F.J.-Z.); 13 UCAM Research Center for High Performance Sport, Universidad Católica de Murcia, 30107 Murcia, Spain; emarin@ucam.edu (E.M.-C.);; 14 Faculty of Sport Sciences, Universidad Católica de Murcia, 30107 Murcia, Spain; 15 Faculty of Sports Sciences and Physical Education, Universidade da Coruña, 15001 A Coruña, Spainmarta.sevilla@udc.es (M.S.-S.); 16 Research Institute of Biomedical and Health Sciences (IUIBS), University of Las Palmas de Gran Canaria, 35001 Las Palmas, Spain; faniya1@gmail.com; 17 Preventive Medicine Service, Centro Hospitalario Universitario Insular Materno Infantil (CHUIMI), Canarian Health Service, 35001 Las Palmas, Spain; 18 Regional Unit of Sports Medicine of Principado de Asturias, Municipal Sports Foundation of Avilés, 33402 Avilés, Spain; 19 FC Barcelona Foundation, 08028 Barcelona, Spain; marta.segu@fcbarcelona.cat; 20 Faculty of Health Science and Wellbeing, University of Vic-University Central of Catalonia, 08500 Barcelona, Spain; 21 Department of Didactics of Language, Arts and Physical Education, Universidad Complutense de Madrid, 28040 Madrid, Spain

**Keywords:** quality of life, lifestyle, well being, weight status, sleep, screen time, children, adolescents

## Abstract

Background: Maintaining a healthy lifestyle is crucial for safeguarding the well-being and quality of life perception, appropriate growth, and development of children and adolescents, while also mitigating the risk of future adult-onset diseases. Objective: To assess associations between perceived quality of life and healthy lifestyle and related outcomes in Spanish children and adolescents. Methods: Cross-sectional analysis of 8–16-year-old children and adolescents (n = 3534) were included in the nationwide study of Physical Activity, Sedentarism, and Obesity in Spanish Youth (PASOS). Data were collected through (1) questionnaires on health-related quality of life (HRQoL), healthy lifestyle outcomes (dietary intake, physical fitness, sleep, and screen time), and (2) anthropometric measurements for weight status assessment. Data were analysed by logistic regression, using the health-related quality of life (HRQoL) as the grouping variable. Results: Participants with a lower HRQoL were those with a lower adherence to the MedDiet and lower achievement of the recommended daily intake of fruit and vegetables. They were also less likely to follow the recommendations for screen time and sleep (with the exception of the weekend) compared to participants with a higher HRQoL. Participants with a lower HRQoL showed a lower healthy weight status and poorer physical fitness than those with a higher HRQoL. Conclusions: Healthy eating habits, healthy weight status (normal weight), appropriate sleep time, physical fitness, and limited screen time play a crucial role in the perceived quality of life in children and adolescents.

## 1. Introduction

The significance of prioritizing the health-related quality of life (HRQoL) in the child and youth population extends far beyond individual well-being, encompassing the broader dimensions of societal health, development, and the fundamental principles of human rights. The child and youth demographic represent not only the present, but also the future of society. Moreover, the World Health Organization (WHO) recognizes the multidimensional nature of health, extending beyond mere absence of disease to encompass physical, mental, and social well-being. Embracing this holistic perspective is paramount in fostering the comprehensive development of children and youths. By prioritizing the health-related quality of life in the child and youth populations, societies not only fulfil a moral imperative but also lay the groundwork for sustainable development. The United Nations Convention on the Rights of the Child (CRC) explicitly outlines the rights of children to the highest attainable standard of health, emphasizing the interconnected-ness of health and human rights [1].

The concepts of “Healthy lifestyle”, “Quality of Life”, and “HRQoL” are interrelated. A healthy lifestyle refers to actions and choices a person makes in life that impact on their own health, including actions such as eating right and being active, keeping body fitness, getting enough sleep, or connecting socially, among others. Quality of life is a broad and subjective concept that changes according to the individual and their circumstances and refers to the general perception of well-being and satisfaction with several aspects of life, such as physical and mental health, social relationships, income level, environment, etc. [2,3]. The World Health Organization (WHO) defines the quality of life as “an individual’s perception of his or her position in life in the context of the culture and value systems in which he or she lives and in relation to his or her goals, expectations, standards and concerns” [4]. It can be affected by several factors such as socio-demographic/economic factors (age, gender, educational level, income, etc.), healthy lifestyle outcomes (diet, physical activity, sleep time, screen time), and environmental factors [5,6]. HRQoL focuses specifically on the perception of quality of life with regard to health. It involves subjective measures of people’s emotional, social, physical, and functional well-being and how these affect their overall well-being and their ability to lead a full and satisfying life.

Being a multidimensional concept, specific questionnaires and instruments are used for assessment. For example, for HRQoL: PedsQL, KIDSCREEN-27; for the social indicators: Child Well-Being Index, KIDSCOUNT Project; and for the subjective well-being: Student Life Satisfaction Scale, Brief Multidimensional Students Life Satisfaction Scale, Personal Well-Being Index-School Children, Subjective well-being measures within the PROMIS framework [1,7]. Hence, these concepts are, therefore, interrelated. Adopting healthy habits/actions can improve physical and mental health, which in turn can positively impact on the overall perception of well-being and life satisfaction. These concepts also differ from each other: quality of life encompasses multiple aspects beyond health, while HRQoL focuses specifically on how health influences the perception of well-being. Adopting a healthy lifestyle has a positive contribution on maintaining or improving the overall health of individuals, their well-being, and, thus, HRQoL [1,2,3,5,6,7].

Research studies have provided evidence that following a healthy dietary pattern, as the Mediterranean Diet (MedDiet), is associated with improved health outcomes [8,9,10]. A high adherence to this dietary pattern was linked to benefits for the psychosocial health of adolescents [8]. Conversely, a poor adherence to the MedDiet increases the risk of cardiovascular disease risk factors, such as insulin resistance, central obesity, and hypertriglyceridemia in school-age children [9]. The relationship between dietary patterns and cardiovascular health highlights the significance of nutritional choices in shaping long-term well-being. In tandem with dietary considerations, the detrimental impact of sedentary behaviours as such screen time on the health of children and adolescents is well-established.

A greater amount of screen time was associated with worse unhealthy dietary patterns, greater consumption of fast food, sweets, and candy; and lower consumption of fruit, vegetables, legumes, dairy products, fish, and nuts [11]. Multiple studies have under-scored the role of reducing screen time as a significant contributor to better health in this population [12,13,14]. Sleep plays a fundamental role in hormonal and metabolic functioning and regulation, and poor sleep time is associated with dysregulation of mechanisms related to hunger and satiety. Yet, sleep patterns have often not been included in global studies related to healthy lifestyles [15].

Physical fitness is improved by performing different kinds of physical activity, so there is sufficient evidence on physical activity and the benefits for people’s health and well-being. Studies have suggested a positive relationship between physical activity and improved psychological well-being in young individuals [13,16]. The correlation between physical fitness and psychological well-being in children aged 10–14 years was assessed. The findings showed that higher levels of physical fitness were linked to a decrease in peer loneliness, as well as a reduction in depressive symptoms. Moreover, improved cognitive, social, and athletic competences were observed, along with increased levels of self-esteem [17]. Muscular fitness and speed/agility were associated with positive effects on bone health [18]. Similarly, fitness was associated with stress, anxiety and HRQoL [19].

Physical fitness was associated with weight status, which means that a poor physical fitness was associated with an increased risk of being overweight or obese [20]. Children living with being overweight or with obesity can enhance their cognitive abilities by promoting physical activity within the school and community; moreover, it has been demonstrated that school-based dietary interventions can be positive on academic performance [17].

Cardiorespiratory fitness was associated with total and abdominal adiposity and cardiovascular risk factors. The findings showed that factors such as the intensity of physical activity and fitness are closely linked to blood lipid levels and other cardiovascular disease risk factors [16,21]. Increasing physical activity levels can decrease blood pressure, blood glucose, and body weight, and improves lipid levels, contributing to the prevention of both initial and recurrent cardiovascular events in individuals of all ages, from young adulthood to the elderly [19].

Previous studies [6,22,23,24,25,26,27,28] analysed the association between HRQoL and healthy lifestyle outcomes, but they were conducted using a regional sample, assessed only one outcome, were not in a national representative wide sample of the Spanish child and adolescent population, and did not assess the association between HRQoL and healthy lifestyle outcomes.

The maintenance of healthy lifestyle in children and adolescents, including optimal weight and fitness, showed clear benefits for health. However, it is crucial to assess how this lifestyle is related to perceived quality of life in young individuals. By understanding this relationship, we can better understand how these behaviours and actions affect their overall well-being and life satisfaction.

The current study aimed to assess associations between perceived quality of life and healthy lifestyle and related outcomes in Spanish children and adolescents.

## 2. Methods

### 2.1. Study Design

A cross-sectional analysis was conducted within the frame of the Physical Activity, Sedentarism, and Obesity in Spanish Youth study (PASOS-2019) undertaken by a national representative, observational, and multicentre research. The details of the PASOS study have been previously published [29].

### 2.2. Participants, Recruitment, Randomization, Data Collection, and Ethics

**Figure 1:**
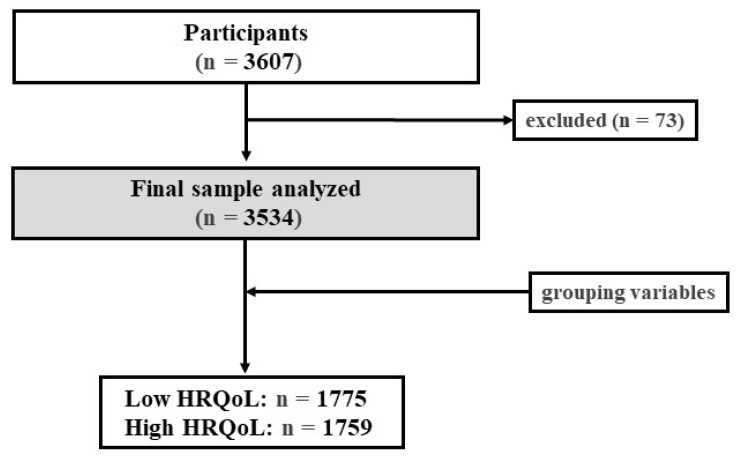
Flow-chart of the study.

The study participants were children and adolescents between 8 to 16 years of age living in Spain. A total of 242 primary and secondary schools (public, private, and subsidised) from the 17 autonomous communities of Spain were randomly selected (ensuring the proportionality of the sample), with a total of 3607 participants. Students with intellectual disabilities that prevented them from answering the questionnaires were excluded. Each case was assessed with the teacher and parents/legal guardians prior to exclusion. The final sample was 3534 children and adolescents because missing data were excluded (n = 73) (Figure 1). The recruitment period was between March 2019 and February 2020, and data collection took place from April to June 2019.

The PASOS study adhered to the principles outlined in the Declaration of Helsinki and received approval from the Ethics Committee of the Fundació Sant Joan de Déu, Barcelona, Spain (ref. PIC 179-18; 17 December 2018). Each participant’s parent or legal guardian provided a signed informed consent form. Only children and adolescents who had given informed consent positively signed by their parents/legal guardians participated in the study. The trial was registered in 2019 with the International Standard Randomized Controlled Trial (ISRCT) under the number 34251612. Further details are accessible at https://doi.org/10.1186/ISRCTN34251612 (accessed on 9 December 2023) [29].

Field researchers completed a one-day training session on the project methodology, organised by the Gasol Foundation, to minimise the coefficient of inter-observer variation.

### 2.3. HRQoL in Children and Adolescents

The field researchers were responsible for the (face-to-face) data collection in the selected schools. Self-reported lifestyle data were registered online at the schools through different and validated questionnaires with the help of trained staff.

The HRQoL of children and adolescents was assessed using the EuroQol-5 Dimensions-5 Levels (EQ-5D-5L) questionnaire validated by the EuroQol Group. This questionnaire is a universal tool for assessing HRQoL [30,31]. It consists of two main components: the EuroQol-5 Dimensions (EQ-5D) descriptive system and the EuroQol (EQ) Visual Analogue Scale (VAS). The descriptive system of the EQ-5D-5L includes five dimensions: mobility, self-care, usual activities, pain/discomfort, and anxiety/depression. Each dimension has five levels ranging from “no problems” to “extreme problems”. Participants are asked to select the statement that best describes their health status for each dimension. This selection results in a one-digit number representing the chosen level for each dimension (5^5^ = 3125). These digits can be combined into a five-digit number that represents the patient’s overall health status (i.e., 1-1-1-1-1 being the best possible health status and 5-5-5-5-5 being the worst health status) [32]. The reliability of the questionnaire in the study sample was over 0.5. The EQ VAS is a vertical visual analogue scale included in the questionnaire. Participants rated their own health on this scale (of 0 to 100), with the endpoints labelled as “The best health you can imagine (100)” and “The worst health you can imagine (0)” [33]. The VAS serves as a quantitative measure of health outcomes that reflects the patient’s personal valuation.

To standardize the assessment of health status using the EQ-5D-5L questionnaire, the EQ-5D-5L Index was calculated as described previously [32,34]. By adhering to this protocol and employing the index values designated for the Spanish population, the researchers obtained the EQ-5D-5L Index for each of the participants, ranging from 1 (perfect health) to negative values (−1) (indicating states of health worse than death).

The grouping variable in the current paper was obtained by scoring the EQ VAS and the EQ-5D-5L Index Value (EQ VAS/100 + EQ-5D-5L Index Value). The EQ VAS was divided by 100 so that both were equally weighted in the results. The two variables were then summed to obtain the grouping variable: HRQoL [6]. The HRQoL variable was divided into two groups using percentile 50 (1.8570): “low HRQoL” was assigned to each participant with an HRQoL equal or inferior to percentile 50, while “high HRQoL” was assigned to participants over percentile 50.

### 2.4. Weight Status Assessment

Measurements of body weight, height, and waist circumference were obtained from the participants following the standardised protocol of the World Health Organisation (WHO) [35]. A SECA 899 (SECA GmbH, Hamburg, Germany) electronic scale was used to measure the body weight of the participants; a SECA 217 (SECA GmbH, Hamburg, Germany) portable stadiometer for height, and a SECA 201 (SECA GmbH, Hamburg, Germany) flexible tape measure to measure waist circumference. Waist measurements were performed two times, and if the difference between both was up to 1 cm, they were measured a third time and in all cases. Body Mass Index (BMI) was calculated and then used to categorize the weight status of participants, following specific cut-off points established in a Spanish percentile table according to age and sex, according to Fernández et al. [36]. Waist-to-height ratio ≥ 0.5 was used as a signal of abdominal fat accumulation, and it was categorized as abdominal obesity [37].

### 2.5. Healthy Behaviour Assessment

The validated KIDMED Index (reliability Kappa coefficient 0.51–0.85) was used to assess the dietary habits of participants [38,39,40]. This score allows participants to be classified as having good adherence to the MedDiet (8 or more points), medium adherence (between 4 and 7 points), or low adherence (3 or fewer points). From this questionnaire, two variables were created: the daily consumption of the total amount of fruit and vegetables, and the compliance of the fruit and vegetables recommendation (at least 4 or more pieces per day). Fruit and vegetables consumption was a relevant goal, as it is a tricky aspect of diets and one of the hardest to achieve [41,42,43,44].

Hours of sleep on weekdays and weekends were measured through the difference in the time of going to bed and the time of waking up. The Sleep Habits Survey is a validated questionnaire with four questions [45], which were registered from participants that met the daily sleep recommendations (weekday and weekend) according to the National Sleep Foundation [46].

Screen times were assessed using questions from the validated SSBQ questionnaire (reliability Kappa coefficient >0.7), focusing on time spent on different screen activities [47]. The variables include the total number of minutes spent on all these activities separated by weekdays and weekends, as well as whether participants achieved the daily screen-time recommendations (less than 120 min/day) proposed by the American Academy of Paediatrics [48].

### 2.6. Physical Fitness

To assess participants perceived physical fitness, the test of the International Fitness Scale (IFIS) validated questionnaire was used (reliability coefficient: 0.68–0.80) [49]. This questionnaire has 5 questions on the general physical condition perceived by the children and adolescents. Answers were registered on a Likert-type scale.

### 2.7. Statistics

The Statistical Package for the Social Sciences version 27.0 (IBM SPSS Statistics for Windows, Version 27.0. Armonk, NY, USA: IBM Corp) was used for statistical analysis. A total of 3534 subjects were analysed. Quantitative variables are shown as mean and standard deviation (SD). Differences between groups were analysed by Student’s *t*-test. For qualitative variables, frequency and percentage were assessed. Chi-square (χ^2^) analysis was performed to assess differences between groups in categorical variables. Results were considered statistically significant if the *p*-value was <0.05. The Odds Ratio was used to analyse the association between the grouping variable (low or high HRQoL) and variables related to diet, sleep, screen time, weight status, and physical activity. A score below 1 indicates a lower probability of occurrence, while a score above 1 indicates a positive association or higher probability; a 95% confidence interval for OR is also presented in the Results. Odds Ratio was calculated twice: the first time without adjustment (crude Odds Ratio), and the second time adjusted by kids’ age, BMI, and gender as potential confounders.

## 3. Results

Table 1 shows the sociodemographic characteristics of the participants according to perceived HRQoL. Children who reported a lower perceived HRQoL and their parents showed a higher age than those with a high perceived HRQoL (*p* < 0.001). Participants with a lower perceived HRQoL showed higher percentages of overweight, obesity, and abdominal obesity, but a lower normal weight than those with a high HRQoL (*p* < 0.001). Participants and their parents with a high perceived HRQoL showed a lower BMI than those with a low HRQoL (*p* < 0.001).

Table S1: Sociodemographic characteristics of parents according to HRQoL of their children.

Table 2 shows the association between perceived HRQoL and lifestyle habits among participants. A low perceived HRQoL was positively associated with a low and moderate adherence to the MedDiet (Cronbach-Alfa form KidMed: >0.35). Participants with a low HRQoL followed fewer the recommendations for fruit and vegetable consumption than those with a high HRQoL. On weekdays, participants with a lower HRQoL slept for fewer sleep hours and met the screen time recommendations less than those with a high HRQoL. A low HRQoL was related to meeting the screen time recommendations less on both weekdays and weekend days. Participants with a low HRQoL spent, on average, 40 min more on screen per day than those with a high HRQoL (Cronbach-Alfa form SSBQ: >0.78).

The association between perceived HRQoL and anthropometric parameters is shown in Table 3. Participants with a high HRQoL showed a high healthy weight status (normal weight) than those with a low HRQoL, which was positively associated with overweight and obese and high abdominal obesity.

Table 4 shows the association between perceived HRQoL and physical fitness in children and adolescents. Participants with a lower HRQoL showed lower physical fitness, expressed as lower general fitness status, cardiorespiratory physical condition, speed and agility, muscle strength, and flexibility (Cronbach-Alfa form IFIS: >0.76).

## 4. Discussion

The main findings of this study were that a high perceived HRQoL in children and adolescents is related to healthy lifestyles, physical fitness, and weight status (normal weight). Those participants who self-reported a high perceived HRQoL had a high adherence to the MedDiet and met the daily recommendations for fruit and vegetable consumption, sleep time, and screen time more frequently than those with a low perceived HRQoL.

Previous research underscored the pivotal role of healthy lifestyle habits (such as following a Mediterranean Diet, adequate screen time and sleep, optimal physical fitness, among others) during childhood and adolescence as key predictors of HRQoL [22]. Building on these findings, the current study showed a positive association between a low adherence to the MedDiet, a well-known healthy diet, and a decrease in perceived HRQoL. This implies that individuals who showed a high adherence to the MedDiet exhibited a tendency to enjoy a high perceived HRQoL than their counterparts with a low HRQoL. This aligns with earlier observations made in Spanish adolescents, where a similar positive association between HRQoL and MedDiet adherence was documented [22,23]. Moreover, the influence of specific dietary components is emphasized, with the consumption of fruits, vegetables, and legumes emerging as notable contributors to a high perceived HRQoL, consistent with previous research highlighting their positive impact [24]. Another study found a positive association between HRQoL and the fruit and vegetable portion of the diet [50]. Similarly, it was pointed out that HRQoL was linked to healthy behaviours, especially those related to diet, except for fruit and vegetable consumption [51]. Similar results were also obtained in Greek adolescents, characterized by a favourable association between HRQoL and MedDiet adherence [52,53]. Beyond the Mediterranean context, findings in Lebanese and Australian adolescents showed that improved HRQoL is linked to an enhancement in diet quality [54,55]. Studies focusing on adult populations with cardiovascular diseases revealed that high scores in both physical and mental health were associated with adopting healthy eating patterns, including adherence to the MedDiet [56]. Similarly, among adults grappling with multiple sclerosis, high HRQoL scores were linked to a stronger commitment to the MedDiet [25]. All these studies yielded similar results than those of the current study, despite the former assessed sample being smaller, and despite the used HRQoL questionnaires, and the nationality of the population was different than those of the current study. These findings emphasize the importance of dietary choices and adherence to the MedDiet in positively influencing the HRQoL across diverse age groups and health conditions.

The current association between HRQoL and healthy behaviours factors, particularly in the realm of screen time and sleep duration, within the child and adolescent population is also supported by previous studies. A low HRQoL has been associated with short sleep periods, poor sleep quality, and delayed sleep onset in young children [57]. The need to consider both device-measured and self-reported sleep measures has been highlighted to better compare outcomes [58]. A previous study conducted in Hong Kong showed that a low HRQoL was linked to high screen exposure with reduced sleep time and physical inactivity [59]. Aligning with these observations, a study focused on Iranian children and adolescents emphasized the substantive and independent influence of both physical activity and screen time on HRQoL, reinforcing the significance of these behavioural aspects [60]. A study conducted in China emphasized the importance of limiting screen time from an early age because it was associated with a negative impact on HRQoL [61]. This observation resonates with findings from Australia, where an equilibrium between increased physical activity and maintaining a reasonable amount of screen time was highlighted to yield noteworthy benefits in terms of socioemotional outcomes but also in HRQoL [62]. Adding to this body of evidence, a study involving Brazilian adolescents contributed an additional perspective by revealing an inverse relationship between HRQoL and screen time in adolescents [63]. Collectively, these findings underscore the pervasive influence of lifestyle factors, particularly screen time and physical activity, on the nuanced landscape of HRQoL in children and adolescents across diverse cultural and geographical contexts.

The current study showed a negative association between participants with a low HRQoL and good physical fitness. It was previously observed that HRQoL was positively related to overall physical fitness and cardiorespiratory condition [26]. In Portuguese adolescents, it was found that those with high HRQoL scores also exhibited high physical fitness, particularly robust cardiorespiratory capacity and muscular strength as assessed through the ALPHA health-related fitness battery [7,64]. Extending this correlation to a younger-age cohort, a study assessing the physical fitness of 4–7-year-old children, utilizing the ALPHA-Fitness battery, highlighted that child with heightened HRQoL exhibited robust physical fitness. This study underscored the significance of muscular strength as the most substantial predictor of HRQoL in boys, while speed and agility emerged as crucial factors for girls [28]. A systematic review concluded that children and adolescents exhibiting a high HRQoL reported good health and high levels of cardiorespiratory fitness and muscular strength, high physical and emotional well-being, as well as healthy relationships with their peers, and an inverse correlation between perceived HRQoL and optimal levels of muscular strength, speed, agility, and flexibility [65]. In other words, the HRQoL decreased proportionally to levels of physical fitness [34].

The current findings show that a lower HRQoL was registered at an older age, as well as a high BMI, overweight and obesity, and abdominal obesity, which agrees with previous studies [27]. A previous study showed a higher decline in all areas of the Paediatric Quality of Life Inventory among older adolescents with overweight or obesity [66], and persistent overweight and obesity in children had a negative impact on future HRQoL into adulthood, influencing both physical and mental health and well-being [67]. It was also pointed out that even in severe obesity in children and adolescents, an intensive one-year lifestyle-focused treatment led to significant long-term improvements in quality of life, suggesting that improvements in HRQoL are related to a high weight loss, even if some weight is regained [68]. It was highlighted that abdominal obesity is a major factor affecting several aspects of children’s lives, including emotional, family, and social aspects, ultimately contributing to a decline in HRQoL [69,70], and that muscle quality plays a significant role in how screen time and abdominal obesity affect the quality of life of schoolchildren [71]. Beyond the individual sphere, the weight status of parents was implicated as a contributory factor, shaping an environment conducive to poor healthy eating and inefficient physical activity [72]. This parental influence, in turn, manifested on the perceived HRQoL of their offspring, thereby emphasizing the interconnectedness of the children-perceived HRQoL with the familial dynamics.

To sum up, perceived HRQoL in children and adolescents is intricately tied to the immediate environment they inhabit. This proximal milieu encompasses lifestyle choices, weight status, and physical fitness. The interplay among these HRQoL related outcomes highlights the significance of a comprehensive understanding of the several factors that contribute to the well-being of children and adolescents. By acknowledging the synergistic impact of lifestyle, weight, and physical fitness levels, deeper insights into dynamics that collectively influence the perceived HRQoL in this demographic group were gained. 

### Strengths and Limitations of the Study

The current study focuses on an area of great importance as its aim was to assess health conditions requiring improvements and design intervention strategies to promote health. This work significantly contributes to the understanding of how the healthy behaviours, physical fitness, and weight status of children and adolescents are related to HRQoL. It is important to highlight that the current study is characterized by having a large and nationally representative sample of the child and adolescent population in Spain, which reinforces the strength and applicability of the results obtained in this analysis. The current study has several limitations. First, the PASOS study is an observational and cross-sectional study, so it cannot provide evidence of causality. Second, all questionnaires were self-reported and, therefore, rely on participants’ perceptions, introducing potential biases such as memory bias, misunderstanding, and social desirability bias. Nonetheless, all questionnaires applied were scientifically validated, and protocols and quality controls were adopted to minimize these biases. Using a precise and standardized methodology, along with validated tools administered by qualified personnel, helps ensured the accuracy of the results.

## 5. Conclusions

Healthy eating habits, healthy weight status (normal weight), appropriate sleep time, physical fitness, and limited screen time play a crucial role in the perceived quality of life in children and adolescents.

## Figures and Tables

**Table 1:** Sociodemographic characteristics of children and parents according to perceived HRQoL.

	Low HRQoL ^§^ (n = 1775)	High HRQoL ^§^ (n = 1759)	*p*-Value ^‡^
Children age (years) *	13.00 (2.36)	12.14 (2.28)	<0.001
Parent age (years) *	45.01 (6.08)	44.10 (6.06)	<0.001
Gender			0.008
Male (n; %)	816 (46.0)	888 (50.5)	
Female (n; %)	958 (54.0)	871 (49.5)	
Children weight status			<0.001
Undernutrition (n; %)	18 (1.0)	27 (1.5)	
Underweight (n; %)	98 (5.5)	110 (6.3)	
Normal weight (n; %)	1124 (63.3)	1266 (72.0)	
Overweight (n; %)	405 (22.8)	285 (16.2)	
Obesity (n; %)	114 (6.4)	59 (3.4)	
Children abdominal obesity			<0.001
No (n; %)	1271 (71.6)	1427 (81.1)	
Yes (n; %)	488 (27.5)	320 (18.2)	
Kid BMI (kg/m^2^) *	21.02 (4.21)	19.68 (3.64)	<0.001
Parent BMI (kg/m^2^) *	25.56 (4.64)	25.19 (4.28)	0.022

* Mean values (SD). Abbreviations: BMI: Body Mass Index; HRQoL: Health-Related Quality of Life; SD: Standard Deviation. ^§^ Grouping variable = (HealthTODAY/100) + (EQ5D5Lindexvalue). The cut-off percentiles were as follows: low HRQoL: ≤1.8570; high HRQoL: ≥1.8571. ^‡^ Differences in prevalence across groups were examined using χ^2^ and Student’s *t*-test.

**Table 2:** Association between perceived HRQoL and lifestyle habits among participants.

		High HRQoL ^§^ (n = 1759)	Low HRQoL ^§^ (n = 1775)
Low adherence MedDiet	Crude OR	1.00 (ref.)	2.03 (1.62–2.55) **
	Adjusted OR	1.00 (ref.)	2.00 (1.58–2.53) **
Medium adherence MedDiet	Crude OR	1.00 (ref.)	1.32 (1.15–1.50) **
	Adjusted OR	1.00 (ref.)	1.26 (1.10–1.44) **
High adherence MedDiet	Crude OR	1.00 (ref.)	0.57 (0.50–0.65) **
	Adjusted OR	1.00 (ref.)	0.60 (0.52–0.69) **
Achieve recommendations of fruits and vegetables	Crude OR	1.00 (ref.)	0.49 (0.40–0.59) **
	Adjusted OR	1.00 (ref.)	0.50 (0.42–0.61) **
Achieve daily sleep recommendation (Weekdays)	Crude OR	1.00 (ref.)	0.66 (0.58–0.76) **
	Adjusted OR	1.00 (ref.)	0.77 (0.67–0.89) **
Achieve daily sleep recommendation (Weekend)	Crude OR	1.00 (ref.)	0.97 (0.85–1.11)
	Adjusted OR	1.00 (ref.)	0.93 (0.81–1.07)
ScreenTime recommendation Weekdays	Crude OR	1.00 (ref.)	0.55 (0.48–0.63) **
	Adjusted OR	1.00 (ref.)	0.70 (0.60–0.81) **
ScreenTime recommendation Weekend	Crude OR	1.00 (ref.)	0.50 (0.42–0.59) **
	Adjusted OR	1.00 (ref.)	0.62 (0.51–0.74) **

Values are Odds Ratio (95%CI). Abbreviations: CI: Confidence Interval. HRQoL: Health-Related Quality of Life; MedDiet: Mediterranean Diet. OR: Odds Ratio. ^§^ Percentiles of the Grouping variable = ((HealthTODAY/100) + (EQ5D5Lindexvalue)). The cut-off percentiles were as follows: low HRQoL: ≤1.8570; high HRQoL: ≥1.8571. OR: Adjusted by kids’ age, BMI, and gender. ** *p*-value < 0.01.

**Table 3:** Association between perceived HRQoL and anthropometric parameters.

		High HRQoL ^§^ (n = 1759)	Low HRQoL ^§^ (n = 1775)
Weight status			
Undernutrition	Crude OR	1.00 (ref.)	0.66 (0.36–1.20)
	Adjusted OR	1.00 (ref.)	2.59 (0.68–9.93)
Underweight	Crude OR	1.00 (ref.)	0.87 (0.66–1.16)
	Adjusted OR	1.00 (ref.)	1.20 (0.81–1.79)
Normal weight	Crude OR	1.00 (ref.)	0.67 (0.58–0.77) **
	Adjusted OR	1.00 (ref.)	0.80 (0.67–0.95) *
Overweight	Crude OR	1.00 (ref.)	1.53 (1.29–1.81) **
	Adjusted OR	1.00 (ref.)	1.22 (1.00–1.50) *
Obesity	Crude OR	1.00 (ref.)	1.98 (1.43–2.73) **
	Adjusted OR	1.00 (ref.)	1.89 (0.49–7.28)
Abdominal obesity	Crude OR	1.00 (ref.)	1.71 (1.45–2.00) **
	Adjusted OR	1.00 (ref.)	1.49 (1.15–1.94) *

Values are Odds Ratio (95%CI). Abbreviations: CI: Confidence Interval. HRQoL: Health-Related Quality of Life; OR: Odds Ratio. ^§^ Percentiles of the Grouping variable = (HealthTODAY/100) + (EQ5D5Lindexvalue). The cut-off percentiles were as follows: low HRQoL: ≤1.8570; high HRQoL: ≥1.8571. Adjusted OR: Adjusted by kids’ age, BMI, and gender. * *p*-value < 0.05. ** *p*-value < 0.01.

**Table 4:** Association between perceived HRQoL and physical fitness.

		High HRQoL ^§^ (n = 1759)	Low HRQoL ^§^ (n = 1775)
General fitness status	Crude OR	1.00 (ref.)	0.29 (0.25–0.34) **
	Adjusted OR	1.00 (ref.)	0.34 (0.29–0.40) **
Cardiorespiratory physical condition	Crude OR	1.00 (ref.)	0.29 (0.25–0.34) **
	Adjusted OR	1.00 (ref.)	0.34 (0.29–0.39) **
Muscle strength	Crude OR	1.00 (ref.)	0.42 (0.35–0.49) **
	Adjusted OR	1.00 (ref.)	0.45 (0.38–0.54) **
Speed and agility	Crude OR	1.00 (ref.)	0.44 (0.38–0.51) **
	Adjusted OR	1.00 (ref.)	0.53 (0.45–0.62) **
Flexibility	Crude OR	1.00 (ref.)	0.55 (0.46–0.66) **
	Adjusted OR	1.00 (ref.)	0.63 (0.52–0.76) **

Values are Odds Ratio (95%CI). Abbreviations: CI: Confidence Interval. HRQoL: Health-Related Quality of Life; OR: Odds Ratio. ^§^ Percentiles of the Grouping variable = ((HealthTODAY/100) + (EQ5D5Lindexvalue)). The cut-off percentiles were as follows: low HRQoL: ≤1.8570; high HRQoL: ≥1.8571. Adjusted OR: Adjusted by kids’ age, BMI, and gender ** *p*-value < 0.01.

## Data Availability

There are restrictions on the availability of data for this trial due to the signed consent agreements around data sharing, which only allow for access to external researchers for studies following the project purposes. Requestors wishing to access the trial data used in this study can make a request to pep.tur@uib.es.

## Supplementary Materials

The following supporting information can be downloaded at: https://www.mdpi.com/article/10.3390/nu15245125/s1, Table S1: Sociodemographic characteristics of parents according to HRQoL of their children.

## Abbreviations

BMI: Body mass index; EQ-5D-5L: EuroQol-5 Dimensions-5 Levels; EQ VAS: EuroQol Visual Analogue Scale; HRQoL: health-related quality of life; MedDiet: Mediterranean diet; OR: Odds Ratio; SD: standard deviations; WHO: World Health Organization.
