# Supplementary material for: Perceived Quality of Life Is Related to a Healthy Lifestyle and Related Outcomes in Spanish Children and Adolescents: The Physical Activity, Sedentarism, and Obesity in Spanish Study
Source: Nutrients. 2023 Dec 16;15(24):5125. doi: 10.3390/nu15245125 (PMC10745413; doi:10.3390/nu15245125)
Supplement: Supplementary file 1 [file nutrients-15-05125-s001.zip › nutrients-2735372-supplementary.pdf]

**Table S1.** Sociodemographic characteristics of parents according to HRQoL of their children.

| | **Low HRQoL** § **(n=1775)** | **High HRQoL** § **(n=1759)** | ***p*-Value** ‡ |
|---|---|---|---|
| Parent Health Perception | | | <0.001 |
| Excellent (n; %) | 86 (4.8) | 152 (8.6) | |
| Very Good (n; %) | 422 (23.8) | 507 (28.8) | |
| Good (n; %) | 976 (55.0) | 840 (47.8) | |
| Poor (n; %) | 151 (8.5) | 117 (6.7) | |
| Bad (n; %) | 10 (0.6) | 7 (0.4) | |
| Parent education level | | | 0.040 |
| University degree (n; %) | 465 (26.2) | 511 (29.1) | |
| General Certificate of Education (n; %) | 341 (19.2) | 301 (17.1) | |
| Vocational Education and Training (n; %) | 389 (21.9) | 336 (19.1) | |
| General Certificate of Secondary Education (n; %) | 233 (13.1) | 220 (12.5) | |
| Primary Education (n; %) | 205 (11.5) | 231 (13.1) | |
| No education (n; %) | 19 (1.1) | 27 (1.5) | |
| Parent employment status | | | 0.311 |
| Working (n; %) | 1266 (71.3) | 1234 (70.2) | |
| Home working (n; %) | 205 (11.5) | 211 (12.0) | |
| Unemployment (n; %) | 134 (7.5) | 142 (8.1) | |
| Student (n; %) | 13 (0.7) | 16 (0.9) | |
| Others (retirement+permanent disability) (n; %) | 40 (2.3) | 22 (1.3) | |
| Parent smoking habit | | | 0.111 |
| Yes (n; %) | 435 (24.5) | 366 (20.8) | |
| Former smoker (0 to +5 years) (n; %) | 428 (24.1) | 424 (24.1) | |
| Never smoker (n; %) | 791 (44.6) | 837 (47.6) | |
| Parent BMI (WHO) | | | 0.171 |
| Underweight (Severe, moderate, mild thinness) (n; %) | 34 (1.9) | 42 (2.3) | |
| Normal weight (n; %) | 771 (43.4) | 760 (43.2) | |
| Pre-obesity (n; %) | 506 (28.5) | 505 (28.7) | |
| Obesity class I (n; %) | 184 (10.4) | 165 (9.4) | |
| Obesity class II (n; %) | 48 (2.7) | 30 (1.7) | |
| Obesity class III (n; %) | 12 (0.7) | 6 (0.3) | |

Abbreviations: BMI: Body Mass Index; HRQoL: Health-Related Quality of Life; SD: Standard deviation. § Grouping variable= ((HealthTODAY/100) + (EQ5D5Lindexvalue)). The cut-off percentiles were as follows: low HRQoL: ≤1.8570; high HRQoL: ≥1.8571. ‡ Differences in prevalence's across groups were examined using $\chi^2$
